# Hypovitaminosis D in Patients with Type 2 Diabetes Mellitus: A Relation to Disease Control and Complications

**DOI:** 10.1155/2013/641098

**Published:** 2013-10-22

**Authors:** Hala Ahmadieh, Sami T. Azar, Najla Lakkis, Asma Arabi

**Affiliations:** ^1^Department of Internal Medicine, Division of Endocrinology, American University of Beirut Medical Center, P.O. Box 11-0236, Riad El-Solh, Beirut 1107 2020, Lebanon; ^2^Department of Family Medicine, American University of Beirut Medical Center, P.O. Box 11-0236, Riad El-Solh, Beirut 1107 2020, Lebanon

## Abstract

*Aims*. This study aims at assessing the relationship between 25 (OH) vitamin D (25-OHD) levels and microvascular complications in patients with type 2 diabetes mellitus (DM2). *Methods*. 136 patients (59 ± 11 years) with DM2 (disease duration 8.6 ± 7 years) participated in this cross-sectional study. Anthropometric data, HbA1c, 25-OHD levels, serum creatinine, and urine microalbumin/creatinine ratio were collected. Dilated retinal exam was performed, and diabetic neuropathy was assessed using the United Kingdom Screening Score. *Results*. Serum 25-OHD correlated negatively with HbA1c (*r* = −0.20,  *P* = 0.049). Mean 25-OHD levels were lower in subjects with diabetic retinopathy compared to those without retinopathy (12.3 ± 5.5 versus 21.8 ± 13.7, *P* < 0.001) and lower in subjects with diabetic neuropathy compared to those without neuropathy (16.4 ± 10.4 versus 23.5 ± 14.5, *P* = 0.004). After adjustment for BMI, diabetes duration, and smoking, 25-OHD was an independent predictor of HbA1c (**β**  −0.14; *P* = 0.03). After adjustment for HbA1c, age, smoking, BMI and disease duration, 25-OHD were independent predictors for diabetic retinopathy: OR 2.8 [95% CI 2.1–8.0] and neuropathy: OR 4.5 [95% CI 1.6–12] for vitamin D < 20 versus vitamin D ≥ 20 ng/mL. *Conclusion*. Low serum 25-OHD level was an independent predictor of HbA1c, diabetic neuropathy, and diabetic retinopathy in patients with DM2.

## 1. Introduction

Diabetes affects more than 300 million individuals in the world with significant morbidity and mortality worldwide [1]. In the United States, it has been estimated that the incidence is about 1 million new cases per year [2]. People with diabetes are at high risk of microvascular complications including diabetic retinopathy, diabetic nephropathy, and diabetic neuropathy, which have bad impact on quality of life and are associated with increased mortality [3–5]. Risk factors for the development of diabetic microvascular complications include glycaemic control, age, diabetes duration, and smoking. Additional risk factors are age at onset of diabetes and genetic factors [4, 6].

In parallel to the increase in the prevalence of diabetes mellitus, there has been a resurgence of vitamin D deficiency worldwide [7, 8]. Immigrants to Europe from the Middle East and Asia carry a high risk for vitamin D deficiency [7].

Vitamin D has traditionally been associated with calcemic activities, namely, calcium and phosphorus homeostasis and bone. However, recent evidence from various lines of research suggested nontraditional roles of vitamin D in human health including cancer, autoimmune, infectious, respiratory, and cardiovascular diseases [9–18]. Hypovitaminosis D has recently emerged as one of the factors contributing to the development of both type 1 and type 2 diabetes mellitus [19–25]. The first report between hypovitaminosis D and glucose intolerance dates back to over 20 years ago when Pietschmann et al. showed that serum 25 (OH) vitamin D (25-OHD) levels were lower in patients with DM2 as compared to nondiabetic controls [26]. Since then, several cross-sectional and case-control studies have shown an association between serum 25-OHD level and DM2 [19–25]. The third National Health and Nutrition Examination Survey (NHANES) data showed a negative relationship between fasting blood glucose and 25-OHD concentrations in healthy white postmenopausal women, and in Mexican American men and women but no similar effect was observed in non-Hispanic black population, suggesting this effect might differ by ethnicity [21]. 

While there is accumulating evidence that hypovitaminosis D is associated with higher prevalence DM2, data on the effect of vitamin D deficiency on diabetes control and complications, namely, microvascular complications are scarce. Suzuki et al. showed in a cohort of 581 Japanese diabetic subjects that serum 25-OHD levels were significantly lower in subjects with diabetic retinopathy compared to those without diabetic retinopathy and in those with microvascular complications compared to those without microvascular complications [27]. Inukai et al. showed that serum 25-OHD levels were decreased in DM2 patients with retinopathy and/or proteinuria when compared with DM2 patients who had no microangiopathy [28]. Both in vitro and in vivo studies showed that vitaminD is a neurotrophic substance but its role in diabetic neuropathic pain is uncertain [29]. Lee and Chen showed that vitamin D supplementation over 3 months improved neuropathic symptoms by 50% in diabetic patients whose vitamin D was deficient at baseline [30].

In this study we assessed the relationship between serum 25-OHD levels and diabetes control and microvascular diabetic complications in patients with type 2 diabetes mellitus presenting to a tertiary care center in Beirut. 

### 1.1. Subjects

All patients with type DM2 presenting to the diabetes clinic at our tertiary care center during the study period extending from November to February were invited to participate. 136 patients with balanced representation with regard to socioeconomic status were enrolled in the study.

In addition, 74 healthy patients visiting the clinic during the same period for nondiabetes medical issues served as controls. 

## 2. Materials and Methods 

### 2.1. Assessments

The following data were collected from all patients at study entry: age (years), gender, duration of diabetes mellitus (years), smoking status, history of hypertension, history of dyslipidemia, systolic and diastolic blood pressure (mm Hg), height (cm), and weight (kg). Body mass index (BMI) (kg/m^2^) was calculated. Sun exposure was estimated by asking the patient about the time spent outdoors during the week preceding enrollment in the study. 

Diabetic neuropathy were evaluated using the validated United Kingdom Screening Score, which is a simple score based on the presence or absence of symptoms and signs of neuropathy obtained from detailed physical exam [31]. 

Patients were referred to the ophthalmology clinic for dilated retinal exam. 

Blood was drawn for 25 (OH) Vitamin D that was measured by RIA using the Immunodiagnostic System Limited, UK. The intraassay and interassay variability were below 10%. Our center participates in the international quality assurance program for vitamin D assays DEQAS (DEQAS London, UK). Serum calcium, phosphorus, and CRP were measured as part of the study protocol. In addition, lab studies as part of routine followup for diabetics, including HbA1c, total cholesterol, HDL, LDL, triglycerides, and urine microalbumin/creatinine ratio were also collected. 

Intake of antidiabetic agents as well as other medications and intake of calcium/vitamin D supplements were also recorded. Patients were excluded from the study if they were taking vitamin D at a dose ≥ 1000 IU/day. 

### 2.2. Ethics

The study was approved by the Institutional Review Board of our institution. All participants signed an informed consent before enrollment in the study. 

### 2.3. Statistical Analyses

Statistical analyses were performed using SPSS version 19. Values are presented as mean ±  SD for continuous variables or number (%) for categorical variables. *P* values < 0.05 were considered significant. 

Pearson's correlations (*r*, *p*) were used to assess the relationship between continuous variables such as 25-OHD level, age, HbA1c, BMI, and diabetes duration. 

Independent *t*-test was used to compare means of continuous variables between 2 categories. The relationship between categories of 25-OHD and diabetic neuropathy and retinopathy was assessed using chi-square. 

Linear regression model was created to assess the relationship between HbA1c and 25-OHD after adjustment for BMI, diabetes duration, and smoking status. The models were built using all enter method with HbA1c as dependent variable and BMI, diabetes duration, and smoking status as well as 25-OHD (entered as a continuous variable) were the predictors.

Logistic regression models were created to assess the relationship between diabetic neuropathy, diabetic retinopathy, and 25-OHD, after adjustment for HbA1c, age, smoking, BMI, and duration of diabetes. The models were built with diabetic neuropathy or diabetic retinopathy as outcome and the other variables as predictors. 25-OHD was used as categorical variable in order to calculate the OR for developing diabetic retinopathy or neuropathy in vitamin D insufficient versus vitamin D sufficient subjects. 

Because only few patients had 25-OHD level > 30 ng/mL, the cutoff value of 20 ng/mL was used to categorize patients into vitamin D sufficient and insufficient groups.

## 3. Results

### 3.1. Baseline Characteristics

Baseline characteristics of patients and controls are shown in Table 1. Both patients and controls had appropriate functional status with good activity of daily living. There was no significant difference in mean age, gender distribution, intake of vitamin D supplements or time spent outdoors between diabetics and controls. Diabetics had higher BMI than controls (30.9 ± 5.2 versus 28.8 ± 4.5, *P* = 0.001), had higher systolic and diastolic blood pressures, and were more likely to be hypertensive (83.7% versus 57.0%, *P* = 0.004) or dyslipidemic (90.4% versus 75.3%, *P* = 0.003) compared to controls. Also diabetics had higher CRP (5.36.5 versus 3.42.2, *P* = 0.002). The mean serum 25-OHD level tended to be higher in controls than in diabetics, but the difference did not reach statistical significance (22.5 ± 12 ng/mL 20.1 ± 12.5 ng/mL). 53% of controls and 60% in diabetics had 25-OHD levels < 20 ng/mL (Table 1). 

In diabetics, the mean duration of diabetes was 8.6 ± 7 years, and the mean HbA1c was 7.9 ± 1.6% without difference between genders. Mean serum creatinine, calcium, and phosphorus were within normal ranges. 60% of the patients had 25-OHD below 20 ng/mL. As expected, women were found to have lower 25-OHD levels when compared to men (18.3 ± 10.4 versus 22.6 ± 14.8, *P* = 0.03). 

When comparing diabetic patients with 25-OHD levels < 20 ng/mL to those with levels ≥ 20 ng/mL, there was no difference in age or diabetes duration between groups (Table 2). 

### 3.2. Bivariate Analysis

#### 3.2.1. Correlations between 25-OHD and Diabetes Complications

Serum 25-OHD correlated negatively with HbA1c (*r* = −0.20, *P* = 0.049). 

**Figure 1:**
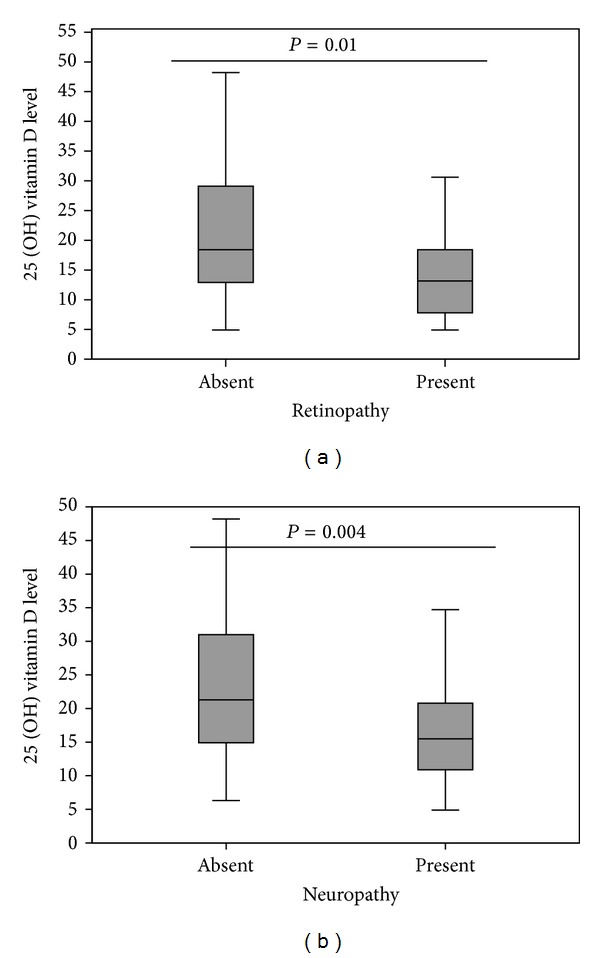
(a)   Box-plot showing the median and 25th and 75th percentiles of 25-OHD values in patients with or without diabetic retinopathy. There was a significant difference between the two groups (*P* = 0.01). (b) Box-plot showing the median and 25th and 75th percentiles of 25-OHD values in patients with or without diabetic neuropathy. There was a significant difference between the two groups (*P* = 0.04).

Mean 25-OHD levels were lower in subjects with diabetic retinopathy compared to those without diabetic retinopathy (12.3 ± 5.5 versus 21.8 ± 13.7, *P* < 0.001) and in subjects with diabetic neuropathy compared to those without diabetic neuropathy (16.4 ± 10.4 versus 23.5 ± 14.5, *P* = 0.004) (Figure 1). Furthermore, using a cutoff value of 20 ng/mL, diabetic retinopathy and neuropathy were more prevalent in subjects with hypovitaminosis D than those with 25-OHD levels ≥ 20 ng/mL: 28% versus 7%, *P* = 0.006 for diabetic retinopathy and 63% versus 42%, *P* = 0.03 for diabetic neuropathy.

Patients with 25-OHD levels below 20 ng/mL had a mean microalbumin/creatinin ratio higher than those with 25-OHD levels ≥ 20 ng/mL (34.5 ± 43.3 mg/g versus 19.9 ± 26.5 mg/g, *P* = 0.04). 

#### 3.2.2. Correlations between 25-OHD and Predictors of Diabetic Complications

25-OHD correlated negatively with age (*r* = −0.19, *P* = 0.05) and with BMI (*r* = −0.2, *P* = 0.04) but not with duration of diabetes. There was no difference in 25-OHD levels between smokers and nonsmokers.

#### 3.2.3. Correlations between Diabetes Complications and Potential Predictors

Patients with diabetic retinopathy were older than those without diabetic retinopathy (62.9 ± 7.8 years versus 58.4 ± 12.2 years, *P* = 0.02). As expected, they reported longer disease duration (10.6 ± 8.1 years versus 7.9 ± 7.0 years, *P* = 0.04) and had higher HbA1c (8.1 ± 1.4 versus 7.5 ± 1.6, *P* = 0.03) than those without retinopathy. There was no difference in BMI between subjects with retinopathy and those without retinopathy. 

 There was no difference in age between patients with and those without neuropathy, but those with neuropathy had longer disease duration and higher HbA1c compared to those without neuropathy (10.9 ± 7.6 years versus 5.5 ± 6.1, *P* < 0.001 for disease duration and 8.3 ± 1.6% versus 7.3 ± 1.3%, *P* < 0.001 for HbA1c).

### 3.3. Adjusted Analyses

After adjustment for BMI, diabetes duration and smoking status, 25-OHD was an independent predictor of HbA1c, with *β* estimate of −0.14 (*P* = 0.03). 

After adjustment for HbA1c, age, smoking, BMI and duration of diabetes in logistic regression model,

- age and 25-OHD levels were significant predictors for diabetic retinopathy with OR of 2.8 [2.1, 8] for 25-OHD < 20 ng/mL versus 25-OHD ≥ 20 ng/mL,
- diabetes duration and 25-OHD were significant predictors of diabetic neuropathy with OR 10.6 [3.5; 15] for duration of diabetes ≥ 7 years versus < 7 years and OR 4.5 [1.6; 12] for 25-OHD < 20 versus 25-OHD ≥ 20 ng/mL.

## 4. Discussion

In this study, low 25-OHD level below 20 ng/mL was highly prevalent in ambulatory patients with type 2 diabetes mellitus. Serum 25-OHD level correlated negatively with HbA1c and was an independent predictor of diabetic retinopathy and neuropathy, after adjustment for potential predictors such as diabetes control, disease duration, age, smoking, and BMI. 

The first published report between hypovitaminosis D and DM2 dates back to over 20 years ago. In 1988, Pietschmann et al. showed that 25-OHD levels were lower in diabetic patients compared to nondiabetic controls [26]. Recently, hypovitaminosis D has emerged as one of the factors contributing to the development of DM2, as many studies showed that patients with DM2 have hypovitaminosis D [19–25]. Need et al. showed that 25-OHD levels were inversely related to fasting serum glucose levels and most markedly when 25-OHD levels were below 40 nmol/l, a level usually associated with secondary hyperparathyroidism, suggesting that the effect of vitamin D on glucose tolerance, if any, is partly mediated through PTH effect [20]. We did not measure PTH levels in this study to assess the relationship between PTH and serum blood sugar level but 25-OHD level in diabetics in our study was less than 20 ng/mL in the majority of patients, thus most of them might have secondary hyperparathyroidism. 

Not only low 25-OHD was highly prevalent in patients with DM2, but also 25-OHD levels were inversely associated with DM2 control assessed by HbA1c value. This is in concordance with the findings in the large population-based Tromsø study where Hutchinson et al. showed a difference in HbA1c of 0.48% between the highest and lowest serum 25-OHD quartiles [32]. 

While there is accumulating evidence that hypovitaminosis D is associated with higher prevalence DM2, data on the effect of vitamin D deficiency on diabetes control and complications, namely, microvascular complications are scarce. In our study, microalbumin/creatinine ratio was higher in subjects with 25-OHD levels below 20 ng/mL when compared to those with 25-OHD levels above 20 ng/mL. Whether this low 25-OHD levels caused diabetic nephropathy or the diabetic nephropathy played a role in decreasing 25-OHD levels cannot be confirmed from the design of the current study. Indeed, low circulating 25-OHD levels have been reported in the majority of patients with renal diseases compared to healthy controls. This was attributed to the urinary wasting of the vitamin D binding protein (DBP) that binds the majority of circulating 25-OHD, which should lead to losses of both vitamin D metabolites. However, 25-OHD has not been consistently detected at increased levels in the urine of patients with renal diseases [33]. 

Serum 25-OHD level was negatively associated with two other diabetic microvascular complications including neuropathy and retinopathy. This relationship persisted even after adjustment for potential confounders. The prevalence of diabetic retinopathy was 24.2%, a percentage that went up to 31.2% in those with 25-OHD levels below 20 ng/mL. 25-OHD level was also lower in patients with diabetic retinopathy compared to those without retinopathy. Few studies have previously shown an inverse relationship between diabetic retinopathy and serum 25-OHD levels [27, 28, 34]. Suzuki et al. showed in a cohort of 581 Japanese diabetic patients that 25-OHD was significantly lower in subjects with 2 or 3 microvascular complications compared to those without complications, namely, in patients with diabetic retinopathy compared to patients without retinopathy [27]. Similarly, Inukai et al. showed that 25-OHD levels were lower and PTH levels were higher in patients with diabetic retinopathy and/or proteinuria compared to those without microangiopathy [28]. Patrick et al. found an association between diabetic retinopathy and prevalence of vitamin D deficiency, but the findings were inconclusive about the existence of a relationship with the severity of retinopathy [34]. 

Because of the cross-sectional design of the current study, we cannot conclude on a causal relationship between low 25-OHD and diabetic retinopathy. Recent evidence indicates possible anti-inflammatory and antiangiogenic properties of vitamin D, as well as its possible role in regulating cell functions such as differentiation, proliferation, and apoptosis in target tissues, including vascular endothelial tissue. Data from animal models support a causal role for vitamin D deficiency in proliferative retinopathy. Indeed, proliferative retinopathy is characterized by neovascularization and angiogenesis and higher serum 1,25(OH)_2_D_3_ reduced angiogenesis in ischemic retinopathy in mice models [35]. On the other hand, the vitamin D receptor (VDR) is present in the human retina, and polymorphisms of VDR gene are related to retinopathy in patients with type 1 diabetes [36]. We did not study genetic risk factors in our study; thus, we cannot exclude the possibility that VDR gene polymorphisms is a confounder for other factors contributing to retinopathy in adults with DM2. 

The prevalence of diabetic neuropathy was 58.5%, a percentage that went up to 67.5% in patients with 25-OHD levels below 20 ng/mL. Also 25-OHD levels were lower in patients with diabetic neuropathy as compared to those without neuropathy. There is emerging evidence that vitamin D is a neurotrophic substance, but its role in diabetic neuropathic pain is uncertain [30]. It has been suggested that vitamin D deficiency may potentiate diabetic nerve damage and may impair nociceptor function, resulting in pain at a threshold of serum 25-OHD higher than that in the nondiabetic population [29]. Lee and Chen showed that vitamin D supplementation over 3 months improved neuropathic symptoms by 50% in diabetic patients with vitamin D deficiency at baseline [30]. 

Our study has some limitations. It is not a population-based study; however, the patients were recruited from a tertiary care center where patients are referred for diabetes care from all over the country, and the population sample was balanced with regard to socioeconomic status. Like all cross-sectional studies, no causal relationship can be inferred.

## 5. Conclusion

In conclusion, low 25-OHD level is an independent predictor of HbA1c, diabetic neuropathy and retinopathy after adjustment for potential confounders in patients with type 2 DM. Therefore, the correction of coexistent low 25-OHD levels may be an important step in the management of DM2 and in the prevention of its complications. Future interventional trials assessing the role of vitamin D substitution in improving the prognosis of diabetic patients are needed.

## Figures and Tables

**Table 1:** Baseline characteristics of diabetic patients in diabetics and controls.

	Diabetics	Controls	*P* value*
Age (years)	59.2 (11.4)	60.1 (10.9)	NS
Male %	38.7%	39.2%	NS
Female %	61.3%	60.8%	
Height (cm)	163.2 (9.6)	162.9 (9.9)	NS
Weight (kg)	83.5 (15.4)	80.1 (11.4)	0.01
BMI (kg/m^2^)	30.9 (5.2)	28.8 (4.5)	0.001
Vitamin D supplementation %	35.9%	36.8%	NS
Systolic BP (mm Hg)	136.1 (22.3)	130.2 (20.4)	0.04
Diastolic BP (mm Hg)	78.7 (12.6)	75.5 (11.9)	0.001
25 (OH) vitamin D (ng/mL)	20.1 (12.5)	22.5 (12)	NS
Calcium (mg/dL)	9.3 (0.4)	9.4 (0.5)	NS
Phosphorous (mg/dL)	3.5 (0.5)	3.7 (0.5)	NS
Creatinine (mg/dL)	0.7 (0.2)	0.8 (0.5)	NS
CRP	5.3 (6.5)	3.4 (2.2)	0.002
Smokers %	51.5%	52.1%	NS
History of hypertension %	83.7%	57.0%	0.004
History of dyslipidemia %	90.4%	75.3%	0.003
25 (OH) D < 20 ng/mL (%)	60%	53%	NS

Values are mean (SD) or percentages, as appropriate.

*P value for difference between groups by *t*-test or chi-square, as appropriate.

**Table 2:** Anthropometric, biochemical characteristics, and parameters of diabetes control in diabetic patients according to 25 (OH) vitamin D level.

	25 (OH) vitamin D < 20 ng/mL (12.4 ± 4.3)	25 (OH) vitamin D ≥ 20 ng/mL (32.6 ± 11.5)	*P* value*
Age (years)	57.6 (11.2)	61.2 (11.1)	NS
Height (cm)	162.0 (9.1)	163.6 (10.5)	NS
Weight (kg)	85.5 (15.1)	79.7 (14.8)	0.03
BMI (kg/m^2^)	31.6 (5.1)	29.6 (5.1)	0.05
Systolic BP (mm Hg)	136.5 (20.7)	135.6 (25.3)	NS
Diastolic BP (mm Hg)	79.2 (13.0)	78.2 (12.0)	NS
Duration of diabetes	8.4 (7.7)	8.8 (6.9)	NS
HbA1c (%)	8.0 (1.6)	7.5 (1.5)	0.04
FBS (mg/dL)	165.5 (65.5)	160.1 (62.1)	NS
Total cholesterol (mg/dL)	179.2 (46.7)	188.1 (66.5)	NS
HDL (mg/dL)	43.4 (11.1)	45.9 (13.9)	NS
LDL (mg/dL)	110.4 (38.9)	109.2 (35.2)	NS
Triglyceride (mg/dL)	175.4 (87.8)	143.5 (59.5)	NS
CRP	5.3 (6.7)	5.0 (6.2)	NS
Creatinine (mg/dL)	0.7 (0.2)	0.7 (0.2)	NS
Microalbumin/creatinin (mg/g)	34.5 (43.3)	19.9 (26.5)	0.04
Calcium (mg/dL)	9.3 (0.4)	9.3 (0.4)	NS
Phosphorous (mg/dL)	3.5 (0.5)	3.5 (0.4)	NS
Retinopathy			
Yes	31.2%	12.0%	0.01
No	68.8%	88.0%	
Nephropathy			
Yes	48.4%	68.2%	0.08
No	51.6%	31.8%	
Neuropathy			0.004
Yes	67.5%	58.0%	
No	32.5%	42.0%	
History of hypertension			
Yes	85.0%	84.0%	NS
No	15.0%	14.0%	
History of dyslipidemia			
Yes	90.0%	92.0%	NS
No	10.0%	8.0%	
Smoking			
Yes	53.8%	66.0%	NS
No	46.3%	44.0%	

Values are mean (SD) or percentages, as appropriate.

*P value for difference between groups by *t*-test or chi-square, as appropriate.

## Abbreviations

DM2: Type 2 diabetes mellitus
25-OHD: Serum 25 (OH) vitamin D
NHANES: National Health and Nutrition Examination Survey
BMIL: Body mass index
HbA1c: Glycosylated hemoglobin
RIA: Radioimmunoassay
PTH: Parathyroid hormone
DBP: Vitamin D binding protein
VDR: Vitamin D receptor.
